# Biomimetic M2 microglia membrane-coated nanoplatform for immune reprogramming and targeted edaravone delivery in ischemic stroke

**DOI:** 10.1093/rb/rbag119

**Published:** 2026-06-13

**Authors:** Yanhong Ren, Peilong Li, Jiaping Li, Sheng Huang, Rutong Yu, Heng Zhao

**Affiliations:** Beijing Institute of Brain Disorders, Laboratory of Brain Disorders, Ministry of Science and Technology, Joint Innovation Center for Brain Disorders, Capital Medical University, Beijing 100069, China; Department of Neurosurgery, Affiliated Hospital of Xuzhou Medical University, Xuzhou 221002, China; Department of Neurosurgery, Affiliated Hospital of Xuzhou Medical University, Xuzhou 221002, China; Department of Ophthalmology, People’s Hospital of Tongren, Guizhou 554300, China; Department of Ophthalmology, People’s Hospital of Tongren, Guizhou 554300, China; Department of Neurosurgery, Affiliated Hospital of Xuzhou Medical University, Xuzhou 221002, China; Beijing Institute of Brain Disorders, Laboratory of Brain Disorders, Ministry of Science and Technology, Joint Innovation Center for Brain Disorders, Capital Medical University, Beijing 100069, China; Department of Ophthalmology, People’s Hospital of Tongren, Guizhou 554300, China

**Keywords:** ischemic stroke, biomimetic microglia, edaravone, oxidative stress, immune reprogramming

## Abstract

Ischemic stroke is a major cause of death and long-term disability worldwide, in which oxidative stress and neuroinflammation contribute substantially to secondary neuronal injury. Edaravone (EDA), a clinically approved free radical scavenger, has shown neuroprotective potential in ischemic stroke; however, its therapeutic efficacy is hindered by a short half-life, limited blood–brain barrier penetration and off-target effects. To overcome these limitations, we developed a biomimetic nanoplatform by encapsulating EDA within a nanoparticle (NP) core and coating it with membranes derived from M2-polarized microglia (EDA@M2 NPs). This hybrid system integrates potent antioxidant activity with inflammation-targeting and immunomodulatory capabilities. In a mouse model of middle cerebral artery occlusion, EDA@M2 NPs selectively accumulated in the ischemic hemisphere, significantly reduced infarct volume, suppressed neuronal apoptosis and improved neurological outcomes. Mechanistically, the nanoplatform not only scavenged excessive reactive oxygen species but also reprogrammed microglia from a pro-inflammatory M1 phenotype toward an anti-inflammatory M2 phenotype, thereby mitigating secondary neuroinflammation. By integrating targeted delivery, oxidative stress attenuation and immune microenvironment remodeling, this combinatorial therapeutic strategy markedly enhanced neuroprotection after ischemic stroke. Our findings demonstrate the translational promise of biomimetic nanotherapeutics for effective ischemic brain injury intervention.

## Graphical abstract

**Graphical Abstract:**
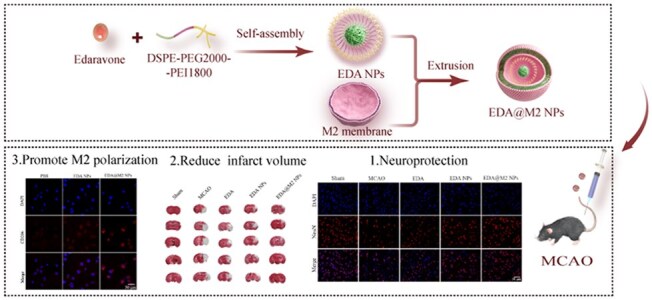

## Introduction

Ischemic stroke is caused by a sudden interruption of cerebral blood flow and remains one of the leading causes of long-term disability and mortality worldwide [1]. Currently, recombinant tissue-type plasminogen activator (rt-PA) is the only thrombolytic agent approved by the US Food and Drug Administration for the treatment of acute ischemic stroke [2, 3]. Its primary therapeutic goal is to rapidly restore cerebral perfusion by dissolving intravascular thrombi [4]. However, the clinical application of rt-PA is limited by a narrow therapeutic time window—typically within 4.5 h of stroke onset—and carries a significant risk of hemorrhagic transformation [5]. These limitations underscore the urgent need for novel therapeutic strategies that can enhance neurological recovery for patients with ischemic stroke.

Neuroprotection is a promising strategy that protects neurons from ischemic injury primarily by inhibiting overactivation of glutamate receptors, overproduction of reactive oxygen species (ROS) and excessive infiltration of inflammatory cells [6–10]. Excessive ROS generation is a key contributor to neuronal injury. After ischemia, brain tissue generates a large amount of ROS, including hydrogen peroxide (H_2_O_2_), superoxide anion (·O^2−^) and hydroxyl radical (^.^OH), as a consequence of ischemia–reperfusion injury [11]. These ROS trigger lipid peroxidation, protein oxidation and DNA damage, leading to neuronal death and blood–brain barrier (BBB) disruption, further exacerbating inflammatory responses and tissue damage [12–14]. Therefore, ROS scavenging is an important strategy to mitigate neurological damage after stroke.

Edaravone (EDA) is a low-molecular-weight free radical scavenger that has been approved for clinical use in the treatment of ischemic stroke [15, 16]. It exerts neuroprotective effects primarily by inhibiting lipid peroxidation and attenuating oxidative stress-induced neuronal injury, thereby mitigating ischemia–reperfusion damage to brain tissue [17, 18]. Additionally, EDA has been shown to indirectly modulate post-stroke inflammation, contributing to improved neurological outcomes [19–21]. However, its clinical utility is hindered by a short plasma half-life, necessitating high-dose administration, which in turn increases the risk of adverse effects such as renal dysfunction [22]. Consequently, the development of an effective targeted drug delivery system is essential to enhance therapeutic efficacy while minimizing systemic toxicity.

Nanodrug delivery systems have been widely investigated in recent years due to their ability to improve targeting specificity, increase drug stability and minimize adverse effects [23–25]. However, the presence of the BBB poses significant challenges to the clinical application of these nanoplatforms, including limited targeting efficiency toward lesion regions, insufficient BBB penetration and potential neurotoxicity in the central nervous system [26, 27]. To overcome these challenges, researchers have been exploring biomimetic nanomaterials based on cell membranes [28]. Among these, macrophage- and microglia-derived membranes are promising coating materials due to their natural targeting and immunomodulatory functions [29]. Particularly, M2 microglia membranes, with anti-inflammatory and tissue-repair properties, can significantly alleviate the inflammatory response and tissue damage after stroke by retaining membrane-associated anti-inflammatory proteins, reducing abnormal infiltration of immune cells, promoting angiogenesis and nerve regeneration [28–33].

**Figure 1:**
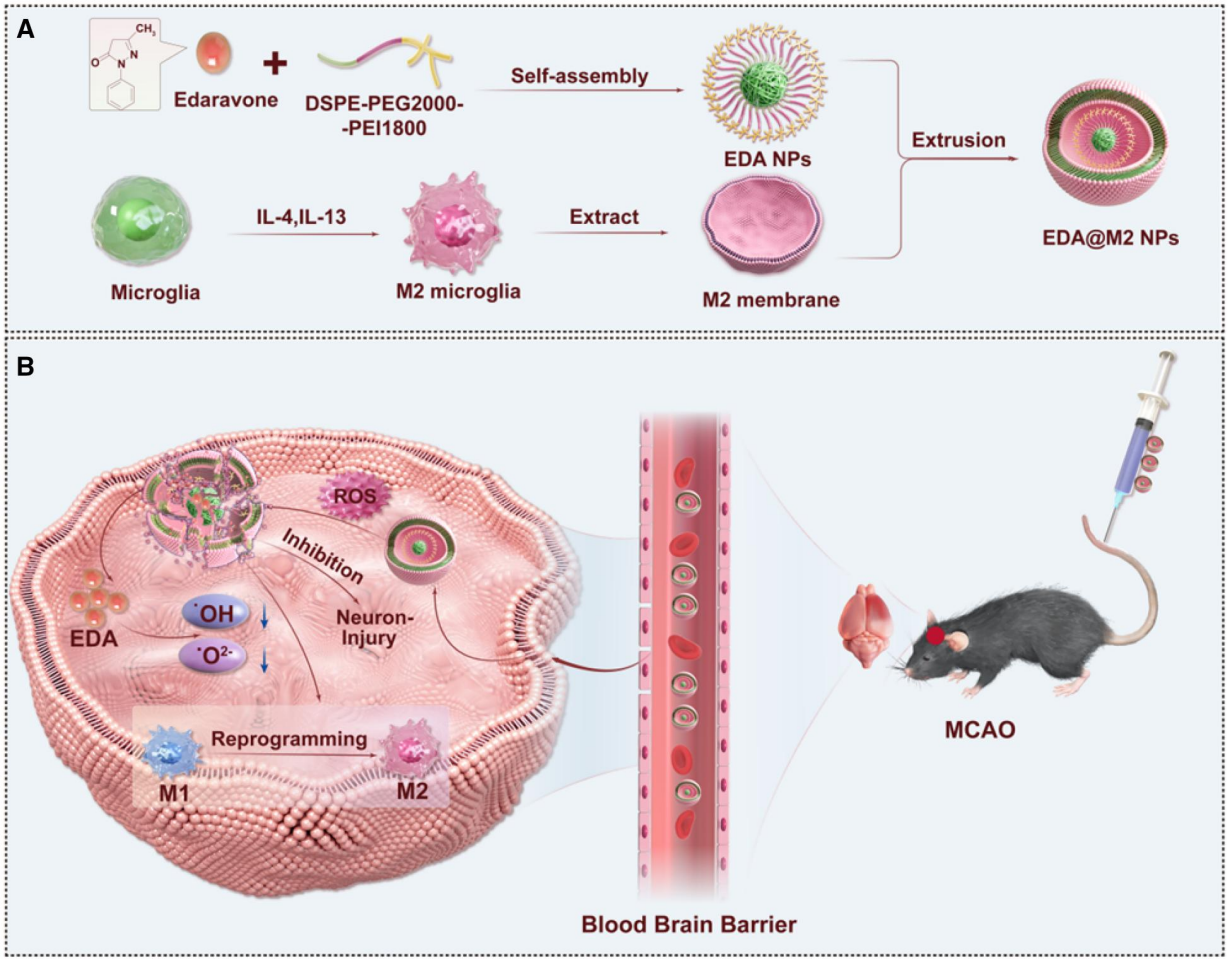
Schematic overview of the EDA@M2 NP system. (**A**) Preparation of EDA@M2 NPs by encapsulating EDA within NPs and coating the NPs with M2 microglia-derived membranes to form a biomimetic delivery system. (**B**) After intravenous administration, EDA@M2 NPs target the ischemic brain lesions via inflammation-targeting properties, enabling enhanced drug delivery across the BBB and accumulation in the infarct area.

In this study, we developed an M2 microglia membrane-based biomimetic nanocarrier [EDA@M2 nanoparticles (NPs)], in which EDA was efficiently encapsulated in the core of the nanocarrier, and specifically delivered EDA to the ischemic regions by taking advantage of the natural targeting and immunomodulatory functions of M2 microglial membranes (Figure 1A and B). M2 microglia membranes not only imitate the biological behavior of M2 microglia but also possess inflammation-targeting capability that can precisely identify and target sites of inflammation in ischemic regions [33, 34]. The EDA released from EDA@M2 NPs was effective in scavenging excessive ROS generated by resident cells (e.g., microglia) as well as infiltrating inflammatory cells (e.g., macrophages and neutrophils) in the brain. Furthermore, M2 microglial membranes retain membrane-associated immunomodulatory proteins that may promote anti-inflammatory signaling and facilitate the polarization of M1 microglia toward the M2 phenotype, thereby synergistically enhancing the neuroprotective effect of EDA.

## Materials and methods

### Materials

DCFH-DA and Cy5 were purchased from MCE (Shanghai, China). Antibodies against CD206, CD86 and Iba1 were obtained from Proteintech (Wuhan, China). The ELISA kit for interleukin-10 (IL-10) was purchased from Beyotime Biotechnology (Shanghai, China), and the TUNEL apoptosis assay kit was obtained from KeyGEN Biotech (Nanjing, China). Recombinant interleukin-4 (IL-4) and interleukin-13 (IL-13) were acquired from NOVO Protein (Suzhou, China). DSPE-PEG2000-PEI1800 was purchased from Xi’an Ruixi Biological Technology Co., Ltd. (Xi’an, China; Cat. No. R-DPEI-038). EDA was purchased from Aladdin (Shanghai, China). Cell culture media, fetal bovine serum (FBS), trypsin and penicillin–streptomycin (P/S) were obtained from Biochannel (Nanjing, China).

### Preparation of M2 microglia membranes

BV2 microglia were purchased from Fuheng Biology (Shanghai, China). BV2 cells were polarized toward the M2 phenotype using recombinant IL-4 (20 ng/mL) and IL-13 (40 ng/mL) for 24 h. The cell suspension was sonicated (2 min, 20 kHz, 30% amplitude) followed by differential centrifugation to isolate subcellular fractions. The resulting supernatant was then ultra centrifuged at 50 000 × g for 30 min at 4°C to collect membrane fractions. The membrane pellet was resuspended in phosphate-buffered saline (PBS) and quantified for protein content using a Bicinchoninic acid assay. Aliquots were snap-frozen and stored at −80°C until use. This method was adapted from previously reported microglia membrane extraction protocols [29, 30].

### Synthesis of EDA NPs and EDA@M2 NPs

To synthesize EDA NPs, DSPE-PEG2000-PEI1800 (20 mg) (Supplementary Figure S1) and EDA (4 mg) were dissolved in a 1:1 (v/v) mixture of chloroform and methanol and subjected to rotary evaporation to form a thin lipid film. The film was rehydrated at 60°C in phosphate buffer and subsequently sonicated (40% amplitude, 30 s).

To prepare EDA@M2 NPs, M2 microglial membranes (2 mg protein) were mixed with EDA NPs (1 mg/mL) and extruded 15 times through a 200 nm polycarbonate membrane using a mini-extruder. This method was adapted from previously reported membrane-camouflaged NP fabrication protocols [28, 31].

### Characterization of EDA@M2 NPs

The morphology of EDA@M2 NPs was visualized using transmission electron microscopy (TEM; Hitachi H7650). The hydrodynamic diameter and polydispersity index (PDI) were measured using a Malvern Nano-ZS90 instrument at 25°C. Zeta potential was also recorded to assess surface charge.

Membrane coating of EDA NPs was confirmed by confocal laser scanning microscopy (CLSM) via co-localization of fluorescein isothiocyanate (FITC)-labeled membranes and Cy5-labeled NP cores. Membrane protein retention was confirmed via sodium dodecyl sulfate–polyacrylamide gel electrophoresis (SDS-PAGE) and Western blot analysis for the M2 marker CD206 and Arg-1.

The stability of EDA@M2 NPs was evaluated by incubating the particles at 37°C under gentle agitation (100 rpm) in PBS (pH 7.4) and 10% FBS for 48 h. The drug loading capacity (DLC) and encapsulation efficiency (EE) were determined by high-performance liquid chromatography (Agilent 1260 Infinity II) at 244 nm, following previously reported NP characterization protocols [23, 24, 35].

DLC and EE were calculated using the following equations:


DLC(%)=(Weight of encapsulated EDA/Total weight of nanoparticles)×100%

EE (%)=(Weight of encapsulated EDA/Total weight of EDA added)×100%.

### *In vitro* cellular uptake and endosomal escape

BV2 cells were seeded into confocal plate at a density of 1 × 10^4^ cells per well and incubated for 24 h. Cy5-labeled EDA@M2 NPs (100 μg/mL) were then added to the culture medium and incubated at 37°C with 5% CO_2_ for 2, 4 or 6 h. After nuclear staining with Hoechst 33 342 (5 μg/mL) for 5 min, excess NPs were removed by washing with cold PBS. Cellular internalization was assessed by CLSM.

To evaluate endosomal escape, BV2 cells were incubated with Cy5-labeled EDA@M2 NPs (100 μg/mL) for 4, 6 or 8 h. Cells were then labeled with LysoTracker Green (50 nM) and Hoechst 33342 (1 μg/mL). CLSM was used to monitor lysosomal co-localization and endosomal escape, according to a previously reported protocol [36].

### *In vitro* cytotoxicity

BV2 microglia and bEnd.3 endothelial cells were seeded in 96-well plates at a density of 1 × 10^4^ cells per well and cultured for 24 h. Cells were then treated with different concentrations of free EDA and EDA@M2 NPs. After 24 h, cell viability was measured using a CCK-8 assay according to the manufacturer’s protocol. This assay was used to evaluate the biocompatibility of the nanoplatform.

### ROS scavenging assay

Intracellular ROS levels were quantified using the redox-sensitive fluorescent probe DCFH-DA. BV2 cells were exposed to H_2_O_2_ (100 μM) for 4 h to induce oxidative stress, followed by treatment with PBS, EDA NPs (100 μg/mL) or EDA@M2 NPs (100 μg/mL). Cells were then incubated with DCFH-DA (10 μM) at 37°C for 30 min. Fluorescence intensity was analyzed by CLSM and flow cytometry (BD FACSCanto II, FITC channel) [14, 30].

### Microglial polarization assays

BV2 microglia were first polarized to the M1 phenotype using lipopolysaccharide (LPS) (100 ng/mL) for 24 h, followed by treatment with PBS, EDA NPs or EDA@M2 NPs for 48 h. After treatment, cells were fixed in 4% paraformaldehyde (PFA), permeabilized with 0.3% Triton X-100 and blocked with 1% bovine serum albumin. They were then incubated overnight at 4°C with primary antibodies against CD206 and CD86 (1:500), followed by Alexa Fluor 594- or 488-conjugated secondary antibodies (1:500). Nuclei were stained with DAPI (1:1000), and images were acquired using CLSM [37, 38].

Cytokine levels in the culture supernatant were measured by enzyme-linked immunosorbent assay (ELISA). After centrifugation (500 × g, 10 min, 4°C) to remove debris, IL-10 levels were quantified by ELISA with absorbance measured at 450 nm.

For protein analysis, cells were lysed after 48 h of treatment (± IL-4) and Western blotting was performed using standard procedures to detect CD206, Arg-1 and β-tubulin as a loading control.

### Mouse middle cerebral artery occlusion/reperfusion model

All animal experiments were approved by the Ethics Committee of Tongren People’s Hospital (Approval No. Tongyi Lun [2025] 74) and were conducted in strict accordance with institutional guidelines and the AVMA Guidelines for the Euthanasia of Animals (2020). Male C57BL/6J mice (8–10 weeks old, weighing 19–22 g) were purchased from GemPharmatech Co. (Nanjing, China). A total of 80 mice were used for different experiments, including infarct volume analysis, immunofluorescence staining, behavioral assessments and *in vivo* biosafety evaluation. For each experiment, mice were randomly assigned to different treatment groups (*n* = 5 per group). The middle cerebral artery occlusion/reperfusion (MCAO/R) model was established following a previously described protocol [39]. Mice were anesthetized with isoflurane, and a silicone-coated monofilament was introduced through the common carotid artery to occlude the MCA for 1 h. Reperfusion was initiated by gently withdrawing the filament. Core body temperature was maintained at 37°C throughout the procedure using a heating pad.

### *In vivo* brain targeting

MCAO mice (*n* = 3 per group) were randomly divided into two groups and intravenously injected with Cy5-labeled EDA NPs or EDA@M2 NPs. Whole-body fluorescence imaging was performed using an *in vivo* imaging system (excitation/emission: 640/680 nm) to monitor NP accumulation in the ischemic brain region [12, 13].

### Evaluation of neuroprotection and microglial modulation

Mice were randomly divided into five groups: Sham, MCAO, EDA, EDA NPs and EDA@M2 NPs. All groups except Sham underwent transient MCAO/R. Immediately after reperfusion, mice were intravenously injected with EDA@M2 NPs at a dose of 4 mg/kg. After 72 h, the mice were humanely euthanized and their brains were collected for 2,3,5-triphenyltetrazolium chloride (TTC) staining, and all efforts were made to minimize animal suffering. Cerebral infarct volume was quantified using TTC staining and analyzed with Image-Pro Plus software.

Neurological function was assessed on days 1, 3, 5 and 7 post-surgery using the Zea-Longa scoring system [0 (normal) to 4 (severe deficit)]. Additional sections were processed for hematoxylin and eosin (H&E) staining and TUNEL assays. Neuronal protection was evaluated by NeuN staining. Microglial phenotype in brain tissue was assessed by immunofluorescence staining for CD86 and CD206, with DAPI used for nuclear counterstaining.

For histological analysis, *n* = 5 mice per group were included. Systematic random sampling was used to select five non-overlapping microscopic fields within the peri-infarct region from each brain section for analysis. All images were analyzed in a blinded manner using ImageJ software. For TUNEL staining, apoptotic cells were quantified by counting TUNEL-positive cells and expressed as a percentage of the total cell count to assess apoptosis levels. H&E staining was used to assess histopathological changes and tissue damage. For Iba1, CD86 and CD206 immunofluorescence staining, quantitative analysis was performed by measuring the mean fluorescence intensity to evaluate microglial activation and M1/M2 polarization.

### Hemolysis assay

To evaluate hemocompatibility, EDA@M2 NPs were incubated with 2% mouse red blood cells (RBCs) in PBS (1:1) at 37°C for 4 h. PBS and deionized water served as negative and positive controls, respectively. After centrifugation (1000 rpm, 5 min), hemolysis was visually assessed by inspecting supernatant color.

### *In vivo* biosafety evaluation

Blood samples were collected for complete blood count and serum biochemical analysis, including liver (ALT, AST, ALB and TP) and kidney (CREA and UREA) function tests. Major organs (heart, liver, spleen, lung, kidney and brain) were fixed in 4% PFA, paraffin-embedded, sectioned at 8 μm and stained with H&E for histopathological evaluation.

### Statistical analysis

All data are presented as mean ± standard deviation (SD). Normality and homogeneity of variance were assessed using the Shapiro–Wilk test and Levene’s test, respectively. Statistical analysis was performed using one-way ANOVA for comparisons among multiple groups. For two-factor experimental designs incorporating a temporal component, statistical analysis was performed using two-way repeated-measures ANOVA. A value of *P* < 0.05 was considered statistically significant. All statistical analyses were performed with GraphPad Prism software (Version 8.0, GraphPad Software, Inc., USA).

## Results and discussion

### Preparation and characterization of EDA@M2 NPs

**Figure 2:**
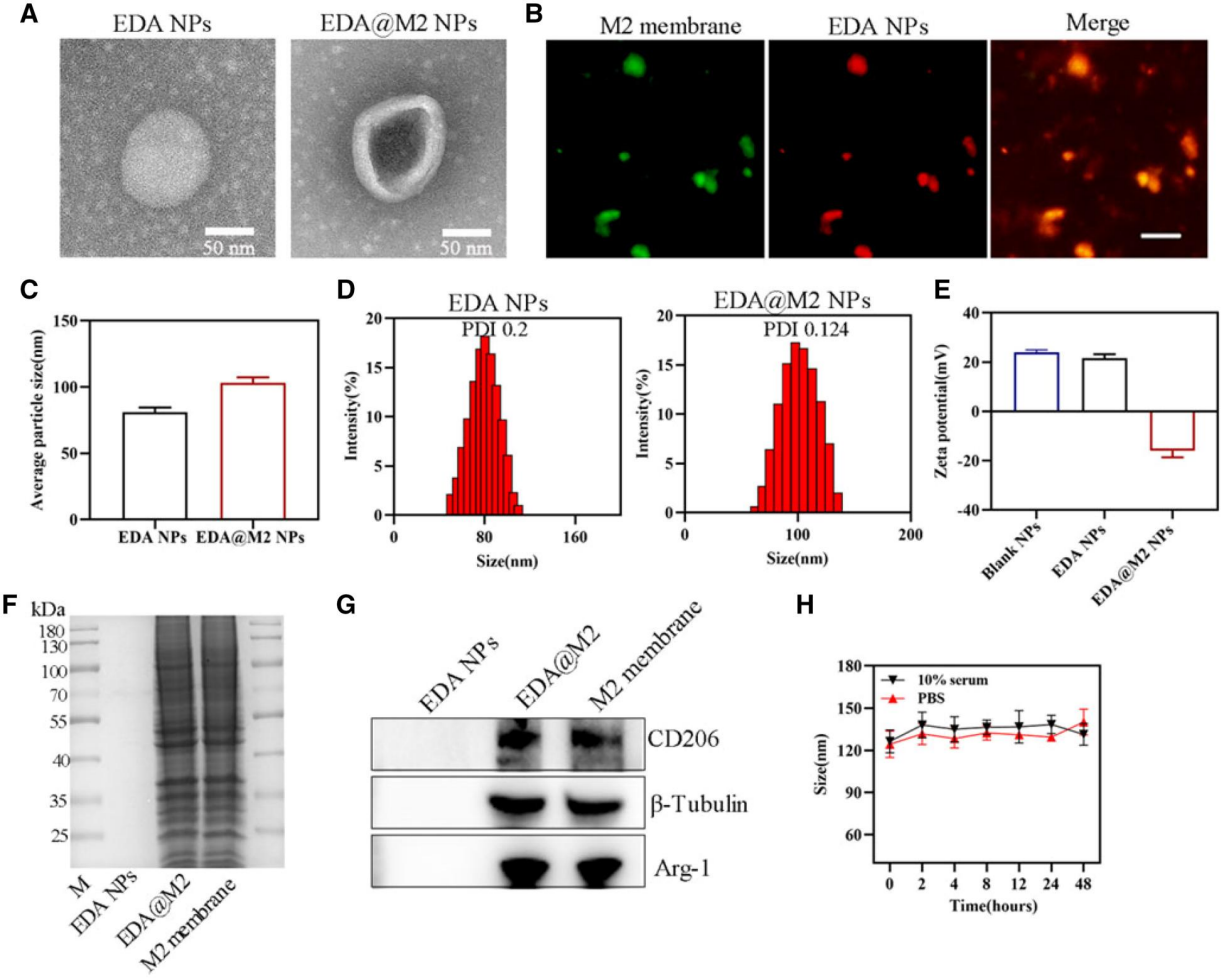
Preparation and characterization of EDA@M2 NPs. (**A**) TEM image showing spherical morphology of EDA@M2 NPs. Scale bar: 50 nm. (**B**) CLSM images showing co-localization of Cy5-labeled EDA NPs and FITC-labeled M2 membranes, indicating successful membrane coating. Scale bar: 10 μm. (**C**) Comparison of average particle size between EDA NPs and EDA@M2 NPs. *n* = 3 per group. (**D**) Size distribution of EDA NPs and EDA@M2 NPs measured by DLS, showing monodispersity, with PDIs of 0.2 and 0.124 for EDA NPs and EDA@M2 NPs, respectively. (**E**) Zeta potentials of blank NPs, EDA NPs and EDA@M2 NPs. *n* = 3 per group. (**F**) SDS-PAGE analysis comparing the protein profiles of M2 membranes and EDA@M2 NPs, with EDA NPs as the control. (**G**) Western blot analysis of CD206 and Arg-1, confirming the presence of M2 membrane proteins on EDA@M2 NPs. (**H**) Stability of EDA@M2 NPs in PBS and 10% FBS over 48 h, shown as size retention. *n* = 3 per group.

The drug delivery system of EDA NPs (EDA@M2 NPs) based on M2 microglial membrane coating was successfully constructed in this study (Figure 2). The morphology of EDA@M2 NPs was characterized using TEM. The results showed that the EDA@M2 NPs exhibited a uniform spherical morphology (Figure 2A). Fluorescence colocalization analysis showed that FITC-labeled M2 membranes (green) showed a high degree of co-localization with Cy5-labeled EDA NPs (red), indicating that the M2 membrane was successfully coated onto the surface of EDA NPs (Figure 2B). This biomimetic membrane coating strategy enhanced the targeting capability of the nanomedicines and thereby improved their delivery efficiency *in vivo* [40].

To characterize the physicochemical properties of the NPs, particle size and zeta potential were measured. Dynamic light scattering (DLS) analysis showed that the hydrodynamic diameter of EDA@M2 NPs was ∼100 nm, and the particle size distribution was narrow. Moreover, the average particle size of EDA@M2 NPs was larger than that of EDA NPs, suggesting that the coating of the M2 cell membrane increased the size of the particles (Figure 2C and D). The NPs possessed a negative surface charge of ∼−18 mV, which may contribute to their colloidal stability (Figure 2E) [41]. The results of Coomassie Blue staining showed that EDA@M2 NPs were highly consistent with M2 cell membranes in protein profiles (Figure 2F), suggesting that membrane proteins were preserved during encapsulation. Western blot analysis further confirmed the presence of the M2 microglial membrane-associated proteins CD206 and Arg-1 in EDA@M2 NPs [42], demonstrating that key functional membrane proteins remained intact after encapsulation (Figure 2G). Compared with the traditional single ligand modification method, this biomimetic envelope strategy can retain the functional proteins of the source cells more effectively, thus enhancing the target recognition ability and biological activity of nanodrugs *in vivo* [43, 44]. To evaluate the long-term stability of EDA@M2 NPs, the particle size distribution at different time points was measured. The results showed minimal size variation after 48 h incubation in PBS (pH 7.4) or 10% FBS, and the EDA@M2 NPs maintained a stable particle size under *in vitro* conditions (Figure 2H), indicating suitability for long-term drug delivery applications [35]. The DLC and EE of EDA@M2 NPs were determined to be 10% and 81.1%, respectively, indicating efficient EDA incorporation within the nanocarrier core.

To further evaluate the release behavior of the nanomedicine, acid conditions were used to simulate the pathological microenvironment of ischemic stroke, and the results were compared with those of free EDA. The results showed that free EDA exhibited rapid release under both physiological conditions (pH 7.4) and acidic conditions (pH 6.5), whereas EDA@M2 NPs exhibited limited drug release under physiological conditions but exhibited significantly enhanced drug release under acidic conditions. These findings indicated that this nanoplatform can effectively enable pH-responsive drug release, thereby reducing premature drug leakage under physiological conditions (Supplementary Figure S2).

### Cellular uptake and cytotoxicity assessment

**Figure 3:**
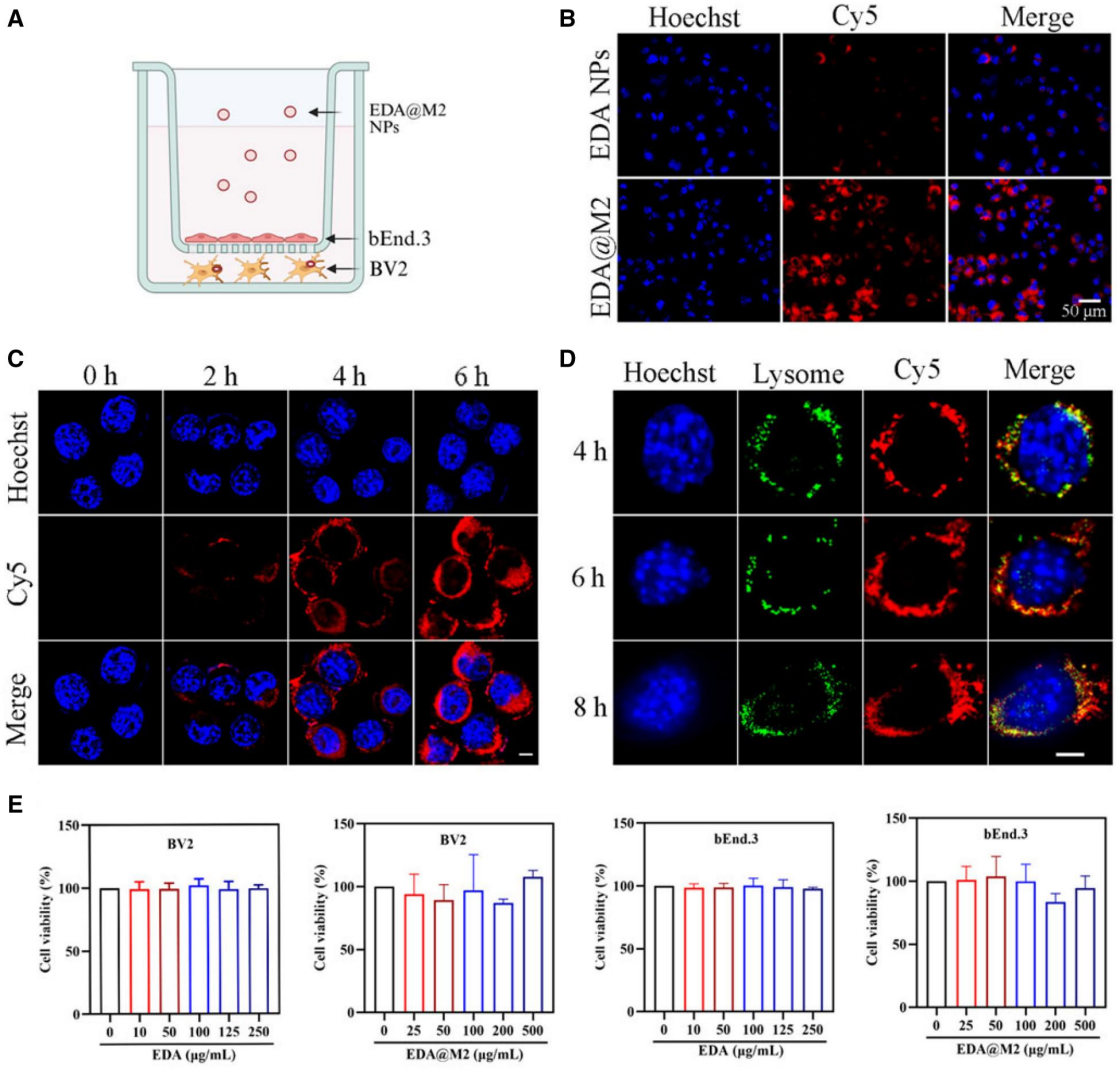
Cellular uptake, lysosomal escape and cytotoxicity assessment of EDA@M2 NPs. (**A**) Schematic illustration of the *in vitro* BBB Transwell model and NP transport across the endothelial monolayer. The schematic was created with BioRender.com. (**B**) Immunofluorescence analysis of BV2 cells in the lower chamber of the Transwell system. Scale bar: 50 μm. (**C**) CLSM images showing time-dependent uptake of Cy5-labeled EDA@M2 NPs in BV2 cells at 0, 2, 4 and 6 h. Scale bar: 5 μm. (**D**) Intracellular colocalization of EDA@M2 NPs and lysosomes in BV2 cells at 4, 6 and 8 h, indicating reduced lysosomal colocalization over time. Scale bar: 5 μm. (**E**) Cell viability of BV2 microglia and bEnd.3 endothelial cells after 24 h incubation with increasing concentrations of free EDA or EDA@M2 NPs, demonstrating negligible cytotoxicity. *n* = 5 per group. Data are presented as mean ± SD.

To evaluate BBB penetration, an *in vitro* Transwell model was established using bEnd.3 cells to form a tight monolayer, which was verified by the transendothelial electrical resistance (TEER) measurements, with BV2 cells in the lower chamber. Cy5-labeled EDA NPs and EDA@M2 NPs were incubated with the Transwell system for 6 h. Cy5 fluorescence intensity in the lower chamber was then measured. As shown in Figure 3B, EDA@M2 NPs showed significantly higher fluorescence intensity in the lower chamber than EDA NPs, suggesting enhanced transendothelial transport. These results indicate that M2 membrane modification facilitates NP transport across the BBB model **(**Figure 3A and B**)**.

To investigate the uptake of EDA@M2 NPs by cells, Cy5-labeled EDA@M2 NPs were co-incubated with BV2 cells for 2, 4 and 6 h. CLSM observations showed that the intracellular Cy5 fluorescence intensity gradually increased with incubation time (Figure 3C, Supplementary Figure S3), suggesting that EDA@M2 NPs were efficiently internalized by BV2 cells in a time-dependent manner.

To evaluate the lysosomal escape capability of EDA@M2 NPs, BV2 cells were incubated with Cy5-conjugated EDA@M2 NPs for 4, 6 and 8 h. Lysosomes were subsequently stained with LysoTracker Green, and subcellular localization was analyzed by CLSM. The colocalization of fluorescence was highest at 6 h, and then decreased significantly at 8 h, suggesting possible lysosomal escape (Figure 3D). This escape behavior is essential for improving the cellular utilization of encapsulated drugs and has been frequently observed in membrane-coated NP systems [36].

To evaluate the potential toxicity of the nanomedicines, different concentrations of free EDA and EDA@M2 NPs were applied to BV2 and bEnd.3 cells, respectively, and cell viability was assessed using the CCK-8 assay after 24 h of incubation. As shown in Figure 3E, cell viability did not change significantly, even at the highest concentrations, indicating that both treatments did not induce obvious cytotoxicity and suggesting a favorable safety profile for further biofunctional studies.

### *In vitro* protective effects of EDA@M2 NPs against oxidative stress

**Figure 4:**
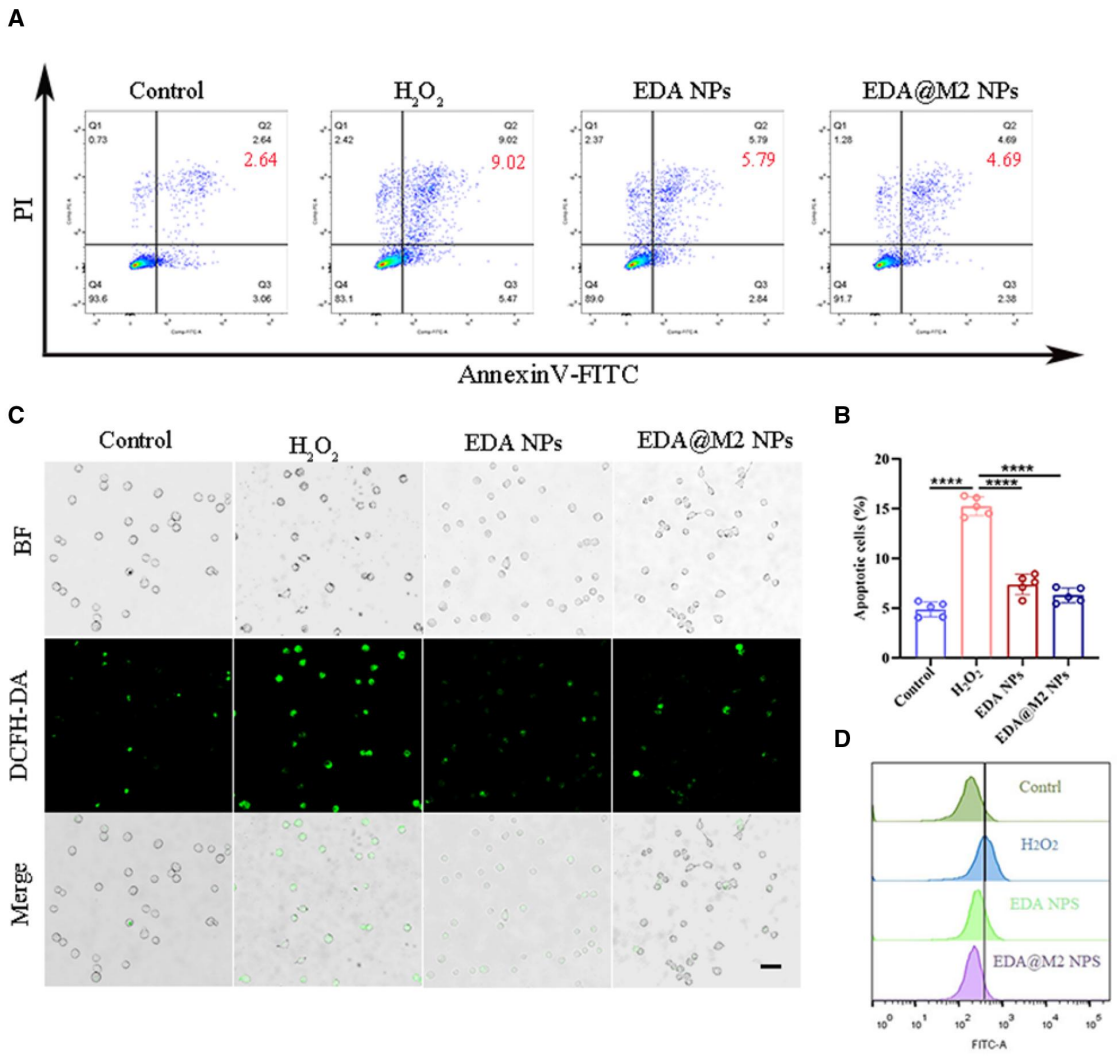
ROS scavenging and anti-apoptotic effects of EDA@M2 NPs in BV2 cells. (**A**) Flow cytometry analysis showing apoptotic cell populations in BV2 cells treated with H_2_O_2_, EDA NPs or EDA@M2 NPs. (**B**) Quantification of apoptotic cell percentages based on Annexin V-FITC/PI staining, showing significantly reduced apoptosis in the EDA@M2 NP group. *n* = 5 per group. Data are presented as mean ± SD. (**C**) Confocal microscopy images of intracellular ROS levels in BV2 cells detected using DCFH-DA staining after various treatments, with green fluorescence indicating ROS accumulation. Scale bar: 10 μm. (**D**) Flow cytometry histograms of ROS-positive cells quantified by DCFH-DA fluorescence intensity, showing the strongest ROS scavenging effect in the EDA@M2 NP group. *P < 0.05, **P < 0.01.

To evaluate the protective effects of the EDA@M2 NPs against oxidative damage, BV2 cells were exposed to H_2_O_2_ and subsequently treated with EDA NPs or EDA@M2 NPs. Flow cytometry analysis showed that H_2_O_2_ markedly increased the apoptosis rate of BV2 cells, whereas treatment with EDA NPs or EDA@M2 NPs significantly reduced the percentage of apoptotic cells. Notably, EDA@M2 NPs exhibited stronger anti-apoptotic effects than unmodified EDA NPs, suggesting superior cytoprotective activity under oxidative stress conditions (Figure 4A and B).

To further assess the intracellular ROS levels, the DCFH-DA probe was used for staining and analyzed by CLSM. The results showed that the percentage of ROS-positive cells in the H_2_O_2_ treated group was significantly increased, whereas it was significantly decreased after treatment with EDA NPs and EDA@M2 NPs, suggesting that both treatments were effective in inhibiting the H_2_O_2_-induced oxidative stress response (Figure 4C). Quantitative flow cytometric analysis of fluorescence intensity showed results consistent with the above findings (Figure 4D).

EDA@M2 NPs can effectively inhibit oxidative stress-induced excessive ROS accumulation and apoptosis, and their antioxidant effect is superior to that of uncoated EDA NPs, which may not only rely on the free radical scavenging ability of EDA itself but also benefit from the synergistic immunomodulatory and antioxidant properties of the nanoplatforms functionalized with M2 microglia membrane [30]. This dual mechanism of “drug-mediated antioxidant and immune-modulating effects” makes EDA@M2 NPs more promising for use in oxidative stress-related diseases, particularly in models of neuroinflammation-related injury.

### EDA@M2 NPs promote the transition of microglia from M1 to M2 phenotype for neuroprotection

To assess the ability of EDA@M2 NPs to modulate microglia polarization in an inflammatory microenvironment, BV2 cells were stimulated with LPS (100 ng/mL) to induce M1 polarization, followed by treatment with PBS, EDA NPs or EDA@M2 NPs. CLSM was used to analyze the fluorescence intensity of CD86 and CD206 [37].

**Figure 5:**
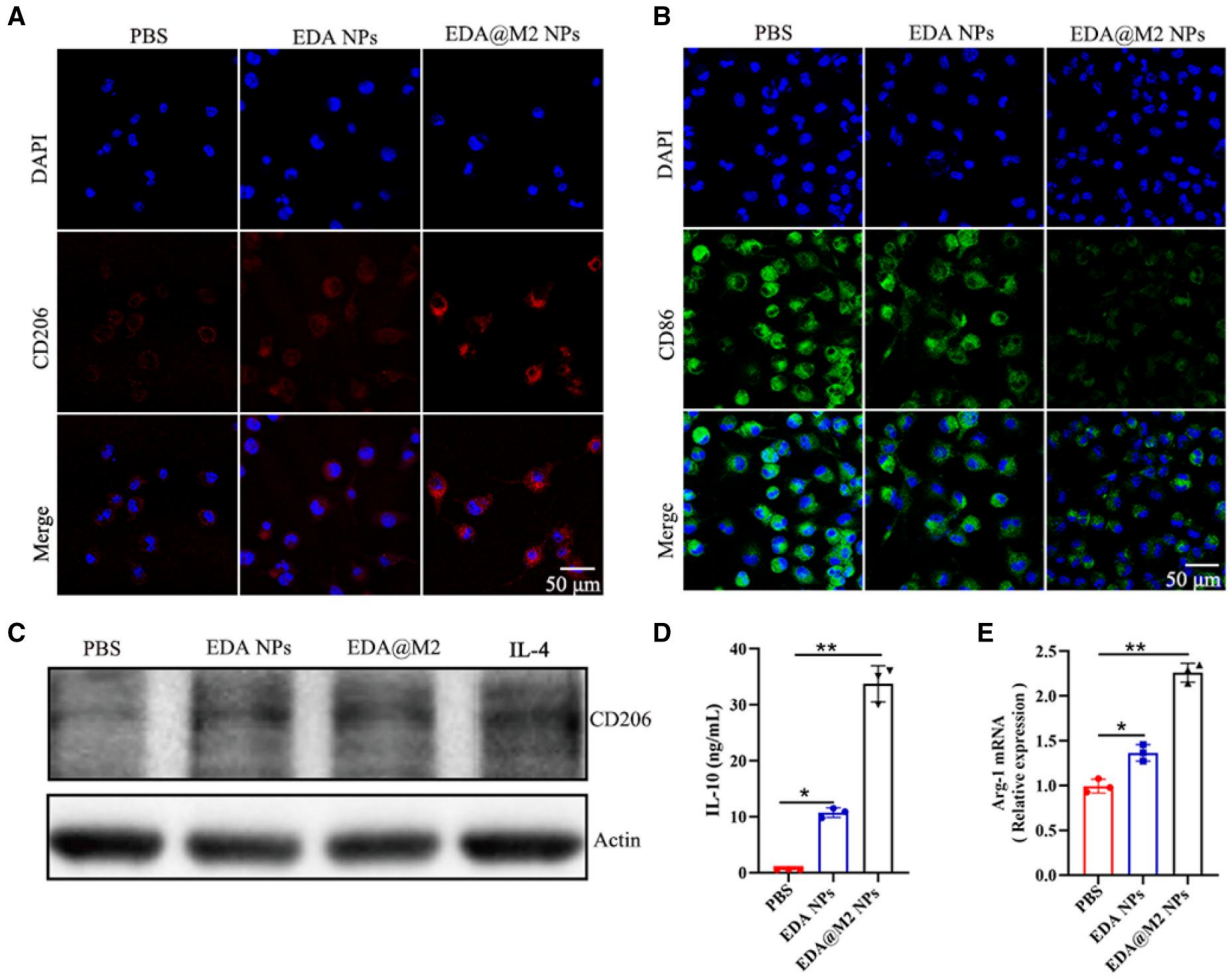
EDA@M2 NPs promote microglial polarization from the M1 to M2 phenotype. (**A**) Confocal microscopy images showing increased expression of the M2 marker CD206 in BV2 cells after treatment with EDA@M2 NPs compared with PBS and EDA NPs. Scale bar: 50 μm. (**B**) Confocal images showing reduced expression of the M1 marker CD86 following EDA@M2 NPs treatment. Scale bar: 50 μm. (**C**) Western blot analysis of CD206 protein expression in BV2 cells after 48 h of treatment. (**D**) ELISA results showing significantly elevated secretion of the anti-inflammatory cytokine IL-10 in the EDA@M2 NPs group. *n* = 3 per group. (E) RT-qPCR analysis of Arg-1 mRNA expression, indicating enhanced M2-type polarization following EDA@M2 NP treatment. *n* = 3 per group. Data are presented as mean ± SD. *P < 0.05, **P < 0.01.

As shown in Figure 5A and B, compared with the PBS group, treatment with EDA@M2 NPs markedly increased CD206 expression and decreased CD86 expression, indicating that EDA@M2 NPs can promote the phenotypic transition of microglia from M1 to M2 (Supplementary Figure S4). These findings suggest that EDA@M2 NPs effectively promoted M2-like polarization of microglia under inflammatory conditions. This result was further verified at the protein level by Western blot analysis (Figure 5C).

In addition, ELISA results showed that EDA@M2 NPs significantly increased the secretion of the anti-inflammatory cytokine IL-10 (Figure 5D). Real-time fluorescence quantitative PCR assay (RT-qPCR) results further confirmed that the expression of Arg-1, a signature gene of M2 microglia, was also significantly increased in this group (Figure 5E). These findings further support the role of EDA@M2 NPs in promoting M2 polarization.

Therefore, EDA@M2 NPs can effectively promote the polarization of microglia to M2 phenotype. The underlying mechanism may involve immune-related membrane proteins (e.g., CD206) and receptors such as the IL-10 receptor present on the M2 microglial membrane, which mimic natural anti-inflammatory signaling pathways in the inflammatory microenvironment and thereby facilitate the restoration of immune homeostasis [38].

In addition, EDA exhibits strong antioxidant activity, synergizing with membrane-derived immunomodulatory effects to attenuate neuroinflammatory responses. Research has shown that M2 microglia are neuroprotective by secreting neurotrophic factors and protecting neurons from damage [38].

### Inflammation targeting and biodistribution of EDA@M2 NPs in MCAO mice

An MCAO mouse model was established to evaluate the targeting ability and *in vivo* biodistribution of EDA@M2 NPs in post-stroke inflammatory regions. Cy5-labeled EDA NPs and EDA@M2 NPs were intravenously administered to mice.

**Figure 6:**
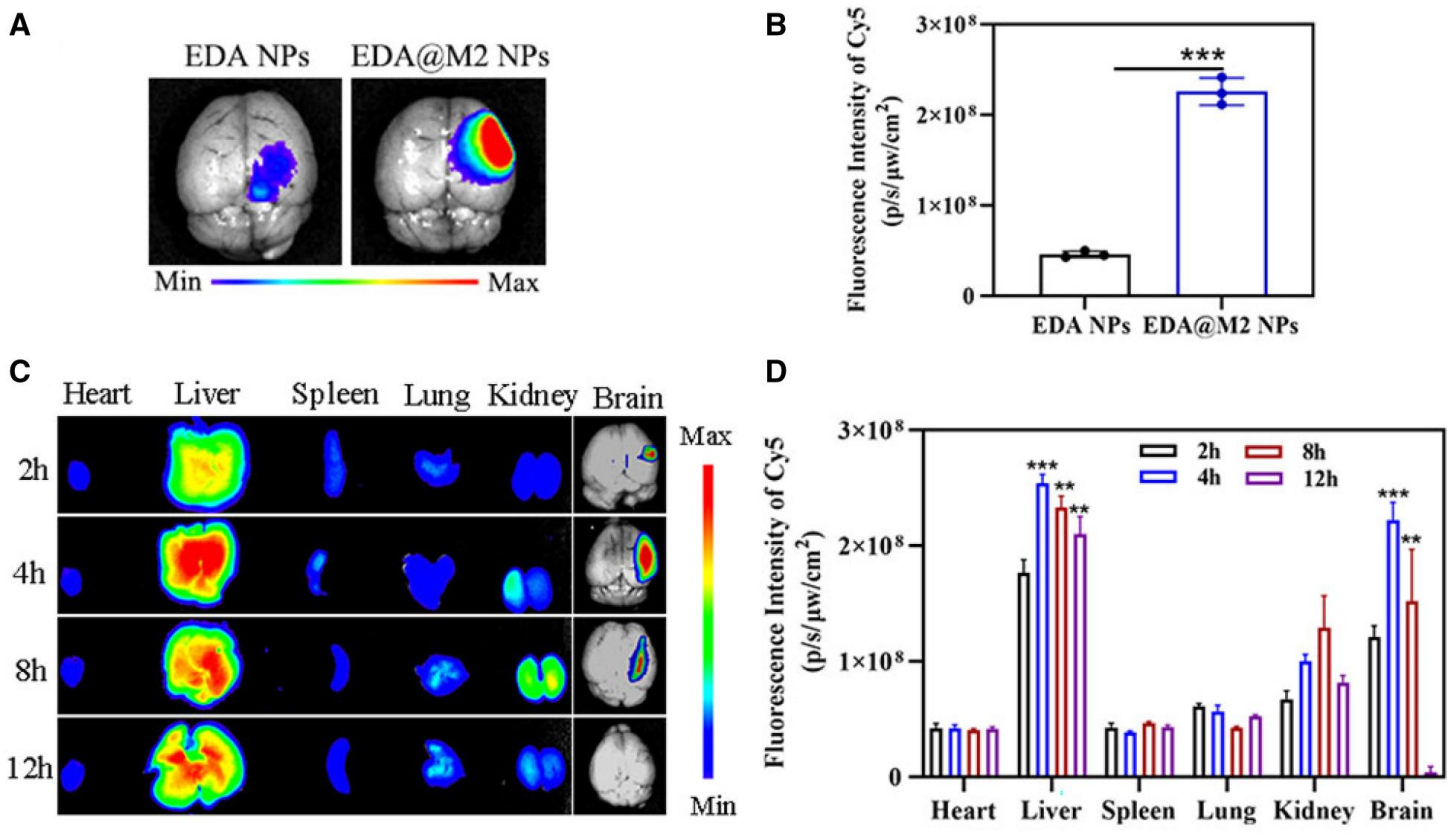
Inflammation-targeting and biodistribution of EDA@M2 NPs in MCAO mice. (**A**) Representative *in vivo* fluorescence images showing enhanced accumulation of Cy5-labeled EDA@M2 NPs in the ischemic brain region compared to EDA NPs. (**B**) Quantification of Cy5 fluorescence intensity in the brain, indicating significantly higher signal in the EDA@M2 NP group. *n* = 3 per group. (**C**) *Ex vivo* fluorescence imaging of major organs (heart, liver, spleen, lung, kidney and brain) at 2, 4, 8 and 12 h post-injection of Cy5-labeled EDA@M2 NPs. (**D**) Quantitative biodistribution analysis showing time-dependent Cy5 signal in various organs, with maximal brain accumulation observed at 4 h. *n* = 5 per group. Data are presented as mean ± SD. **P < 0.01, ***P < 0.001.

Fluorescence imaging showed that EDA@M2 NPs exhibited significantly stronger accumulation in the ischemic brain region compared with unmodified EDA NPs (Figure 6A and B), indicating enhanced lesion-targeting capability. Further immunofluorescence staining revealed that, compared with EDA NPs, Cy5-labeled EDA@M2 NPs colocalized with CD31-positive endothelial cells and Iba1-positive microglia in the ischemic region. These results suggest that the NPs may enter microglia via endothelial transport and accumulate within the ischemic region, thereby enhancing their ability to target the lesion site (Supplementary Figure S5). To further investigate biodistribution, major organs including the heart, liver, spleen, lung, kidney and brain were collected at 2, 4, 8 and 12 h post-injection for *ex vivo* fluorescence imaging. EDA@M2 NPs showed a time-dependent accumulation in brain tissue, with maximal accumulation observed at 4 h, followed by a gradual decrease thereafter (Figure 6C and D). It has been reported that M2 microglia possess the ability to migrate to and accumulate in lesion sites during neuroinflammation [45]. In this study, NPs were coated with M2-type microglial membranes to mimic their intrinsic homing ability, thereby achieving efficient targeted accumulation in ischemic brain tissue.

### *In vivo* therapeutic effects of EDA@M2 NPs on ischemic stroke

**Figure 7:**
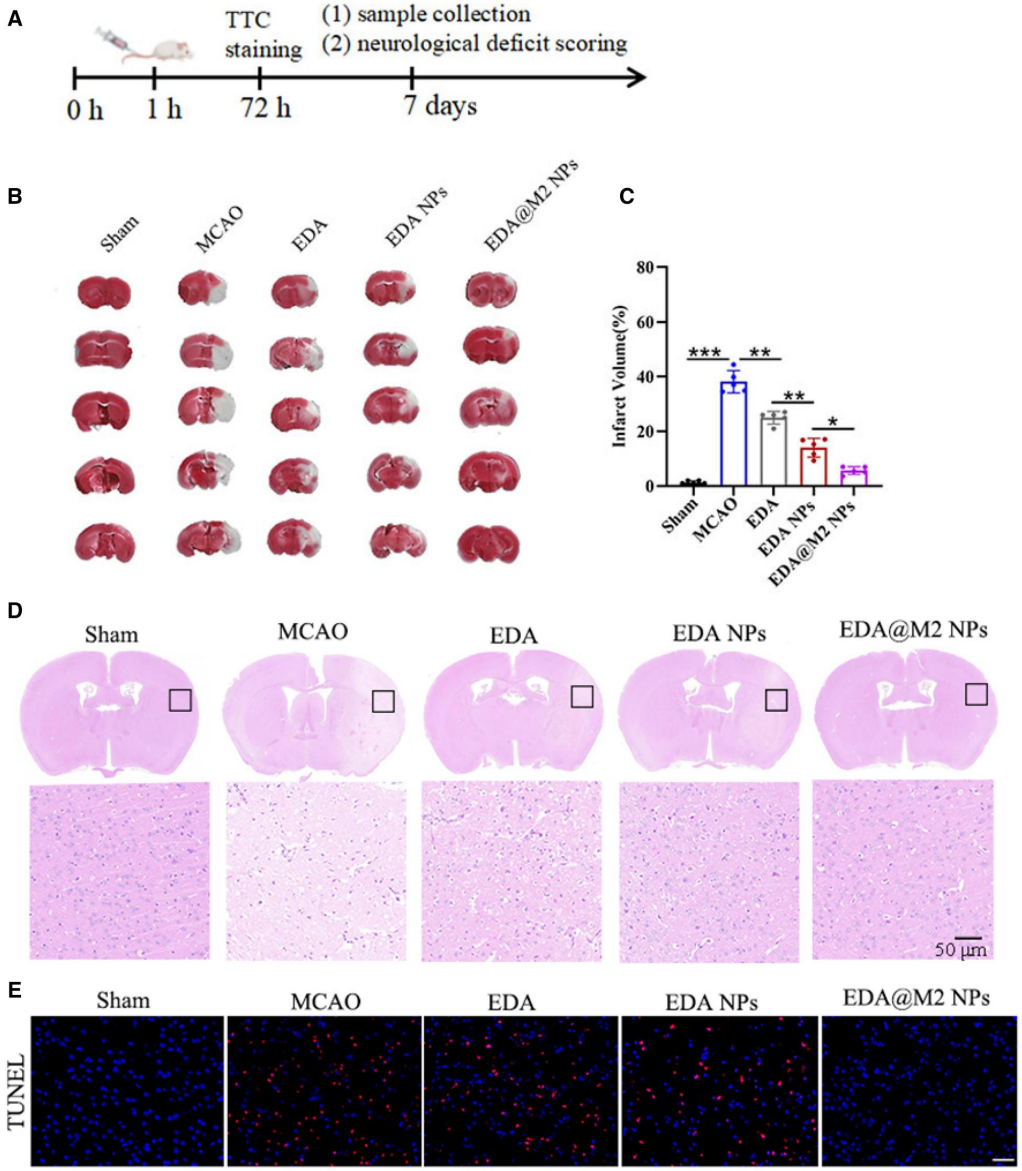
*In vivo* neuroprotective effects of EDA@M2 NPs in a mouse MCAO model. (**A**) Schematic of the experimental timeline showing the MCAO procedure, treatment administration and assessment at 72 h and 7 days. (**B**) Representative TTC-stained brain slices from each group showing infarct volume at 72 h post-ischemia. (**C**) Quantification of infarct volume determined by TTC staining, showing significant reduction in the EDA@M2 NPs group. *n* = 5 per group. Data are presented as mean ± SD. (**D**) Representative H&E-stained brain sections displaying histopathological changes in the ischemic cortex. EDA@M2 NPs markedly attenuated tissue damage. Scale bar: 50 μm. (**E**) TUNEL staining showing apoptotic cells and counterstained nuclei, with reduced apoptosis observed in the EDA@M2 NPs group. Scale bar: 50 μm. *P < 0.05,**P < 0.01,***P < 0.001.

To evaluate the neuroprotective effect of EDA@M2 NPs on ischemic stroke, MCAO mice were treated with PBS, EDA, EDA NPs or EDA@M2 NPs. At 72 h after surgery, the brains were collected, coronally sectioned at 1-mm intervals and subjected to TTC staining (Figure 7A). The results showed that the cerebral infarct volume was significantly smaller in the EDA@M2 NPs treatment group compared with the PBS group, indicating that EDA@M2 NPs exerted significant neuroprotective effects against ischemic injury (Figure 7B and C).

To further assess pathological changes, H&E staining and TUNEL assays were performed. EDA@M2 NPs markedly reduced vacuolization and the number of TUNEL-positive cells, indicating a potent anti-apoptotic effect (Figure 7D and E, Supplementary Figure S6). These findings support the hypothesis that the regulation of neuroinflammation and inhibition of neuronal apoptosis are essential mechanisms in reducing post-ischemic damage [46].

Collectively, these results demonstrate that EDA@M2 NPs exhibit robust neuroprotective effects *in vivo* by alleviating cerebral infarction and reducing neuronal apoptosis.

### EDA@M2 NPs regulate microglial polarization in post-stroke inflammation

EDA@M2 NPs have been shown to effectively induce the polarization of microglia from the pro-inflammatory M1 phenotype to the anti-inflammatory M2 phenotype *in vitro*, indicating their potent immunomodulatory capability. Microglia tend to adopt a pro-inflammatory M1 phenotype, and reversing the polarization state of microglia by microglial reprogramming strategies can effectively attenuate the inflammatory response of brain tissue and promote neuroprotection [47].

**Figure 8:**
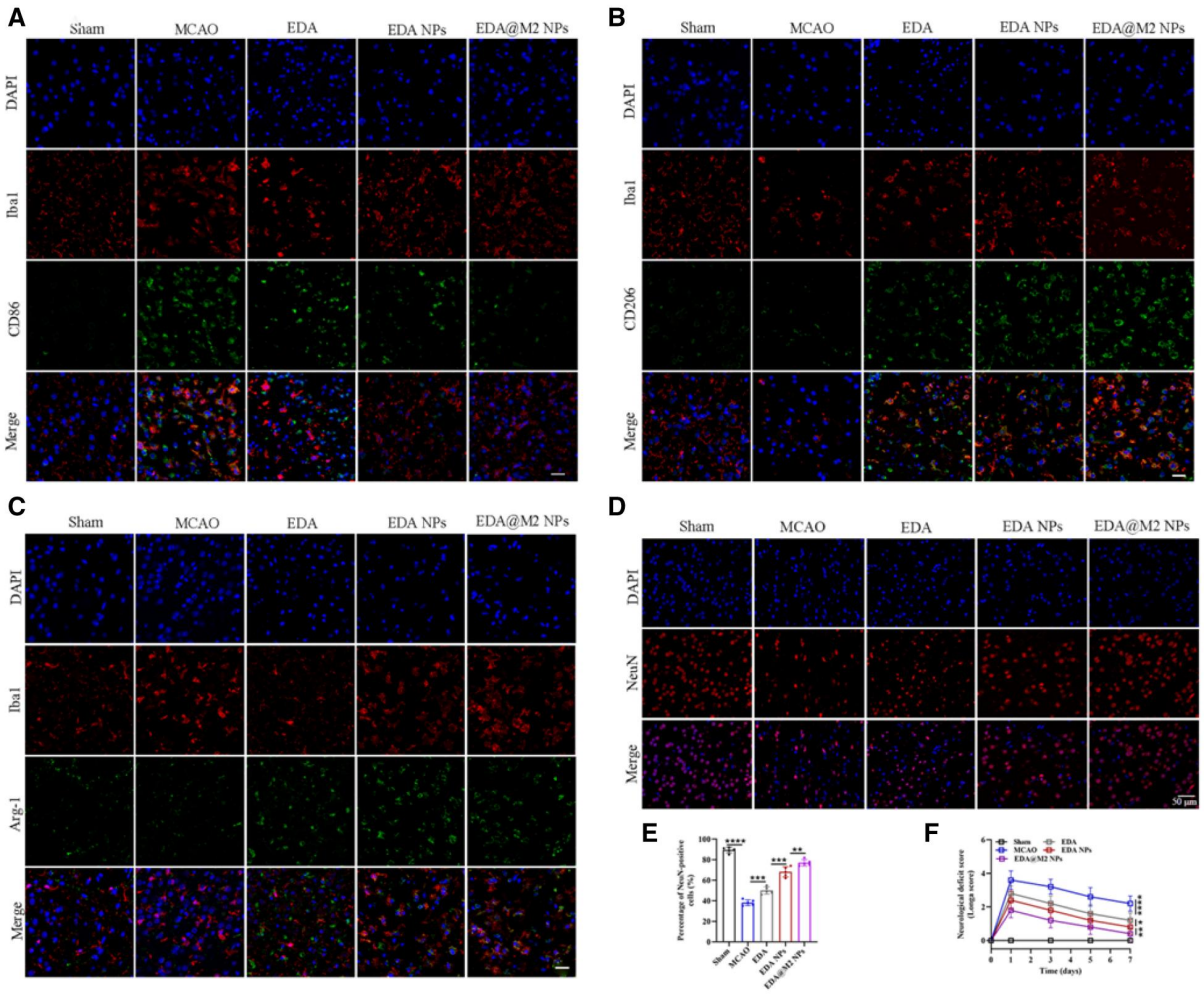
EDA@M2 NPs reprogram microglial polarization from the pro-inflammatory M1 phenotype to the anti-inflammatory M2 phenotype *in vivo*. (**A–C**) Representative immunofluorescence images of brain sections showing co-localization of the microglial marker Iba1 with the M1 marker CD86 (A), M2 marker CD206 (**B**), and M2 marker Arg-1 (**C**). Nuclei were stained with DAPI, and merged images indicate microglial phenotypic changes after treatment. Scale bar: 50 μm. (**D**) Representative immunofluorescence images of NeuN-positive neurons and DAPI-stained nuclei in brain sections from the Sham, MCAO, EDA, EDA NPs, and EDA@M2 NPs groups. Scale bar: 50 μm. (**E**) Quantification of NeuN-positive neurons was performed by counting NeuN-positive cells. *n* = 5 per group. (**F**) Neurological function scores measured at days 1, 3, 5, and 7 after MCAO, demonstrating improved neurological recovery in mice treated with EDA@M2 NPs. *n* = 5 per group. Data are presented as mean ± SD. **P < 0.01, ***P < 0.001, ****P < 0.0001.

To further verify the immunomodulatory ability of nanomedicines *in vivo*, MCAO model mice were treated with PBS, EDA, EDA NPs or EDA@M2 NPs and immunofluorescence staining was performed on their brain tissue sections to evaluate microglial activation and phenotypic polarization. The expression of the pro-inflammatory M1-type marker CD86 was significantly reduced, while the expression of the anti-inflammatory M2-type markers CD206 and Arg-1 was significantly upregulated in the brain tissues of the EDA@M2 NPs group (Figure 8A–C, Supplementary Figure S7). These markers were mainly distributed in Iba1-positive microglia, suggesting that EDA@M2 NPs significantly promoted the phenotypic transformation of microglia from M1 to M2. To further evaluate the neuroprotective effects of EDA@M2 NPs, NeuN immunofluorescence staining revealed that, compared with the PBS group, both the EDA group and the EDA NP group partially preserved NeuN-positive neurons. The EDA@M2 NPs group exhibited significantly higher NeuN fluorescence intensity. This result demonstrates the most pronounced neuroprotective effect of EDA@M2 NPs and indicates that they can effectively promote neuronal survival following ischemic brain injury (Figure 8D–E). In addition, neurological deficits were evaluated using the Zea-Longa scoring criteria. Mice treated with EDA@M2 NPs exhibited significantly lower neurological scores than those in the PBS group, further confirming the neuroprotective potential of EDA@M2 NPs (Figure 8F). This observation is consistent with previous reports that effective inhibition of neuroinflammation and cell death significantly improves the prognosis of neurological function after stroke [46].

To evaluate the delivery performance and therapeutic efficacy of the biomimetic nanoplatform under acute treatment conditions, a single-dose administration strategy was employed in this study. However, in clinical practice, EDA is typically administered twice daily. Therefore, a single-dose regimen cannot fully mimic the sustained drug exposure achieved by repeated administration and may not adequately reflect its long-term therapeutic effects. Accordingly, the long-term efficacy of this system still requires systematic evaluation through repeated dosing regimens in future studies.

Taken together, these results demonstrate that EDA@M2 NPs can effectively reprogram microglial polarization *in vivo*, thereby reshaping the post-stroke immune microenvironment and contributing to the attenuation of neuroinflammation and the promotion of neural repair.

### *In vivo* safety evaluation of NPs

**Figure 9:**
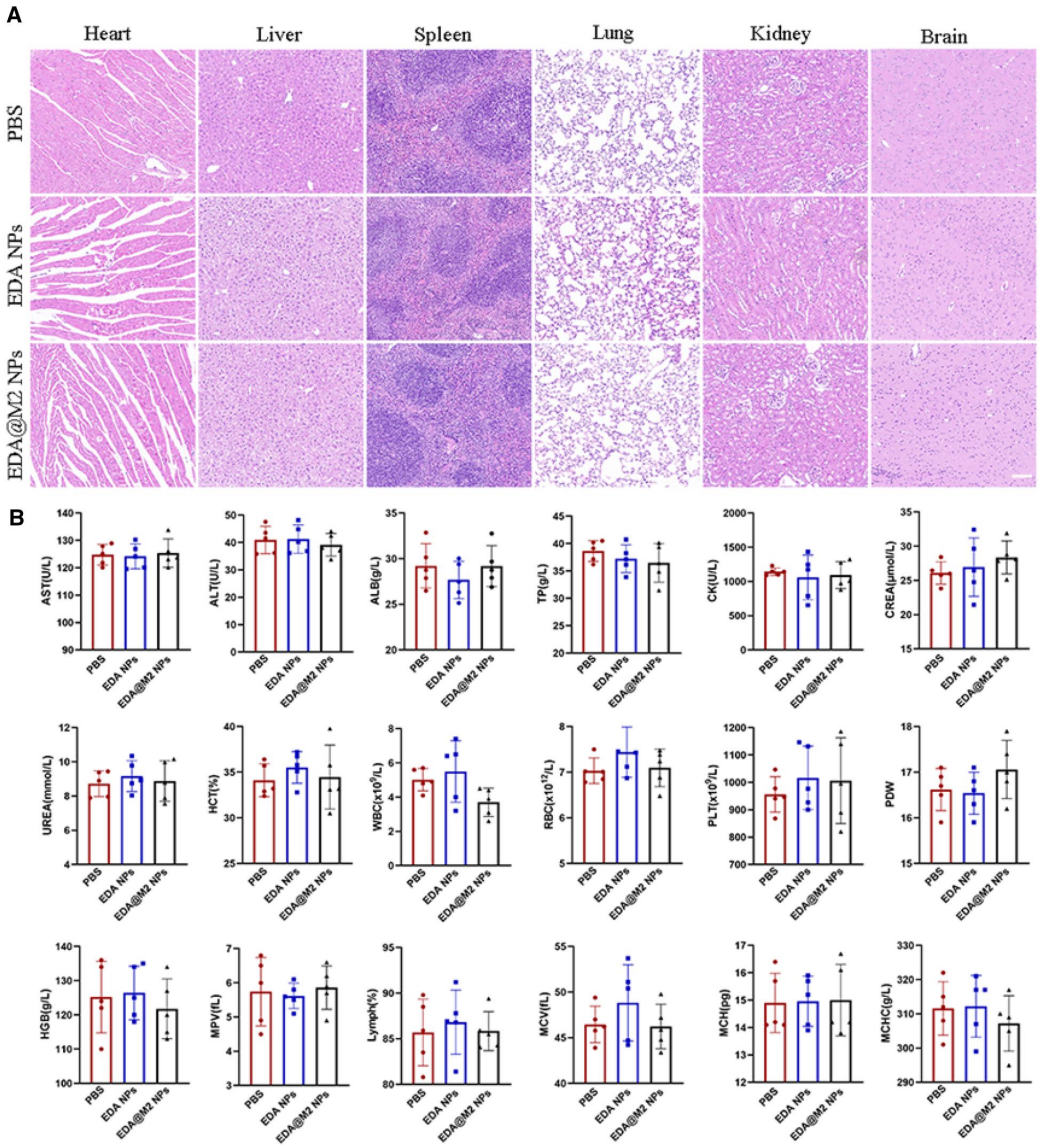
*In vivo* biosafety evaluation of EDA@M2 NPs. (**A**) Representative H&E-stained histological images of major organs (heart, liver, spleen, lung, kidney and brain) from MCAO mice treated with PBS, EDA NPs or EDA@M2 NPs, showing no obvious histopathological abnormalities. Scale bar: 50 μm. (**B**) Serum biochemistry and hematological analyses, including liver and kidney function markers (AST, ALT, ALB, TP, CK, CREA, UREA) and complete blood count parameters (HCT, WBC, RBC, PLT, PDW, HGB, MPV, MCV, MCH, MCHC). No significant differences were observed across treatment groups. *n* = 5 per group. Data are presented as mean ± SD.

The safety of NPs is essential for clinical translation. An ideal nanomedicine delivery platform should not only exhibit excellent pharmacodynamic properties but also possess good biocompatibility and *in vivo* safety [48]. Therefore, we systematically evaluated the safety of this nanomedicine for *in vivo* application by H&E staining and detecting blood biochemical indicators. H&E staining showed that no obvious histopathological abnormalities were observed, and no infiltration of inflammatory cells was observed in the heart, liver, spleen, lungs, kidneys and brain of the mice in each group (Figure 9A). Additionally, RBC, white blood cell as well as liver and kidney function markers in serum biochemical tests showed no significant differences among groups (*P* > 0.05) (Figure 9B). The results of hemolysis experiments showed that the EDA@M2 NPs did not induce a significant hemolytic reaction (Supplementary Figure S8). The hemolysis assay further demonstrated the excellent hemocompatibility and biocompatibility of EDA@M2 NPs. These findings also suggest that the biomimetic membrane coating may contribute to reducing potential biological toxicity.

Collectively, these findings demonstrate that EDA@M2 NPs exhibit no evident acute toxicity *in vivo* and possess favorable biosafety profiles for potential application in ischemic stroke therapy.

## Conclusion

In this study, we developed a biomimetic NP delivery system (EDA@M2 NPs) by functionalizing EDA-loaded NPs with M2 microglia membranes, providing an innovative therapeutic strategy for ischemic stroke. The system exerts its therapeutic effect through a collaborative multidimensional mechanism. Firstly, the M2 microglia membrane coating endowed the NPs with ischemia-targeting ability and enhanced BBB penetration, which allows for precise accumulation at the lesion site. Secondly, the controlled release of EDA effectively scavenges excess ROS from the neuroinflammatory microenvironment. In addition, membrane-derived functional proteins drive the polarization of microglia toward an anti-inflammatory phenotype, thereby restoring neuroimmune homeostasis. Animal experiments demonstrated that EDA@M2 NPs significantly reduced cerebral infarct volume and improved neurological deficits without significant hepatic or renal toxicity. The proposed biomimetic carrier-assisted therapeutic strategy constitutes a scalable paradigm for targeted central nervous system therapeutics, with significant implications for translational applications in diverse neurological diseases.

## Supplementary Material

rbag119_Supplementary_Data

## Data Availability

All data supporting the findings are available upon reasonable request.
