# Supplementary material for: Biomimetic M2 microglia membrane-coated nanoplatform for immune reprogramming and targeted edaravone delivery in ischemic stroke
Source: Regen Biomater. 2026 Jun 13;13:rbag119. doi: 10.1093/rb/rbag119 (PMC13375268; doi:10.1093/rb/rbag119)
Supplement: rbag119_Supplementary_Data [file rbag119_supplementary_data.docx]

Supplementary Information


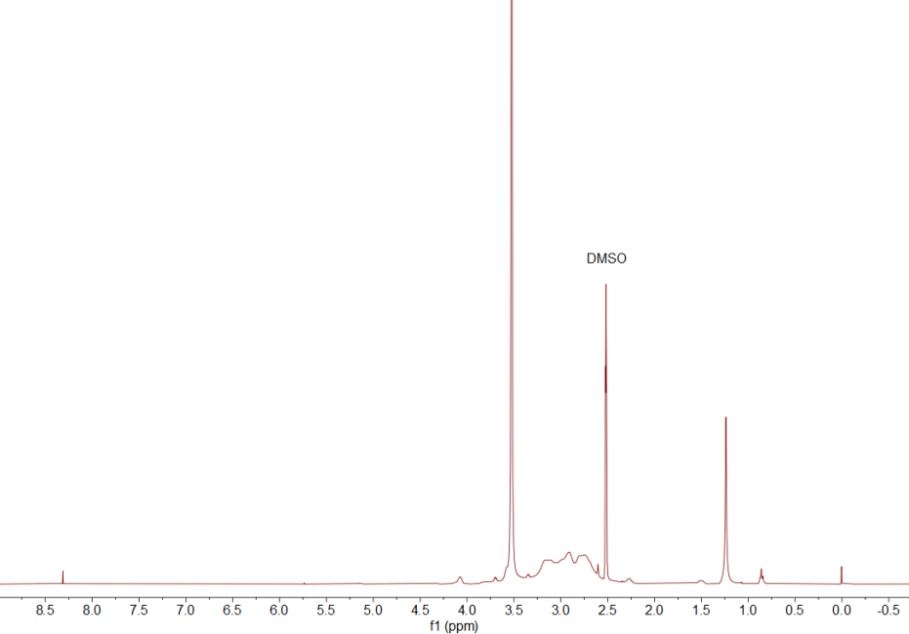


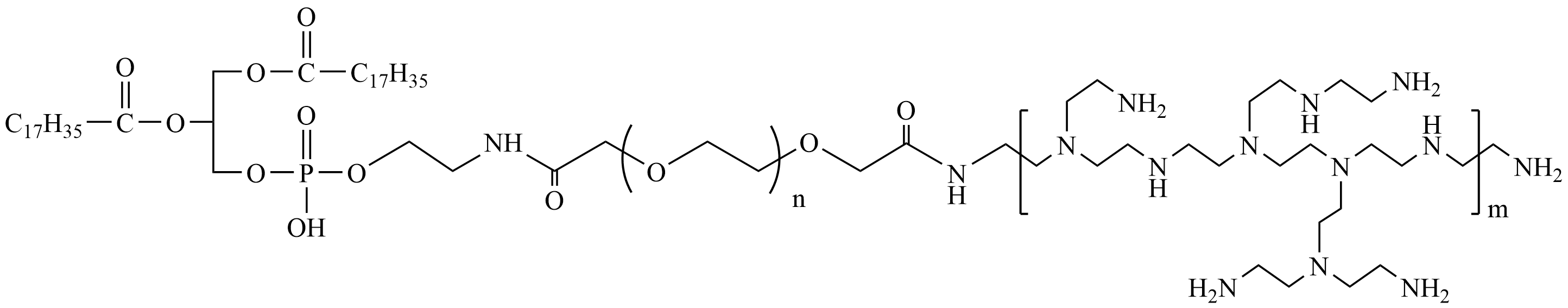


**Figure S1.** ^1H NMR spectrum of DSPE–PEG₂₀₀₀–PEI₁₈₀₀ in DMSO.


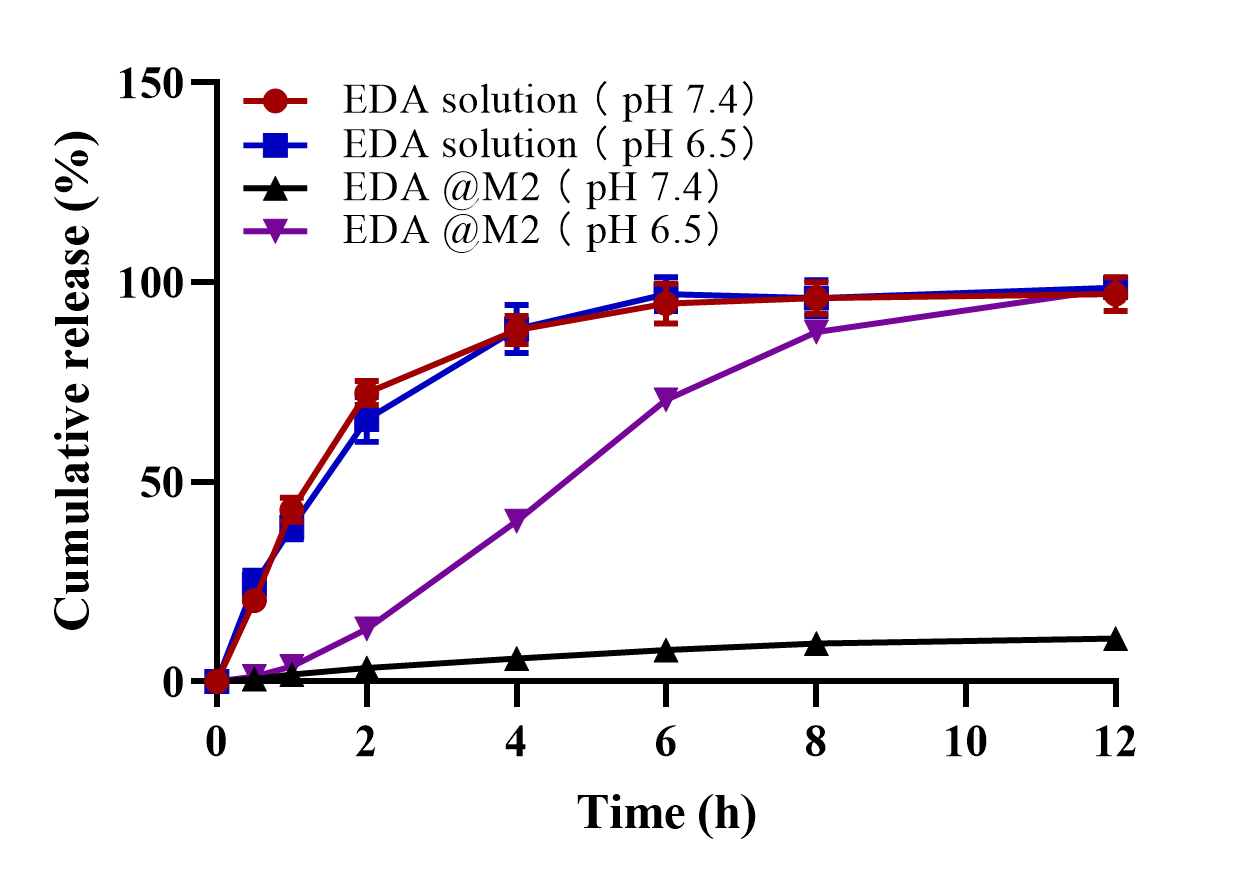


**Figure S2.** In vitro release profiles of free EDA and EDA@M2 NPs under physiological conditions (pH 7.4) and acid conditions (pH 6.5).


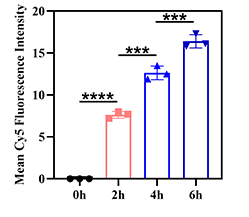
**Figure S3.** Quantitative analysis of mean Cy5 fluorescence intensity in BV2 cells following incubation with Cy5-labeled EDA@M2 nanoparticles for 0, 2, 4, and 6 h. Fluorescence intensity increased in a time-dependent manner, indicating progressive cellular uptake. n = 3 per group. ***p < 0.001, ****p < 0.0001.


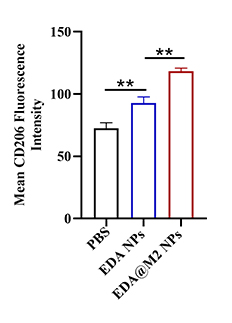

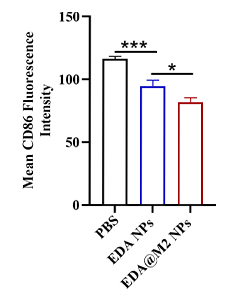
**Figure S4.** Quantitative analysis of mean fluorescence intensity (MFI) of CD86 (left) and CD206 (right) in BV2 cells after treatment with PBS, EDA NPs, or EDA@M2 NPs. EDA@M2 NPs significantly decreased CD86 expression and increased CD206 expression compared to controls, indicating microglial polarization toward the M2 phenotype. n = 5 per group. *p < 0.05, **p < 0.01, ***p < 0.001.


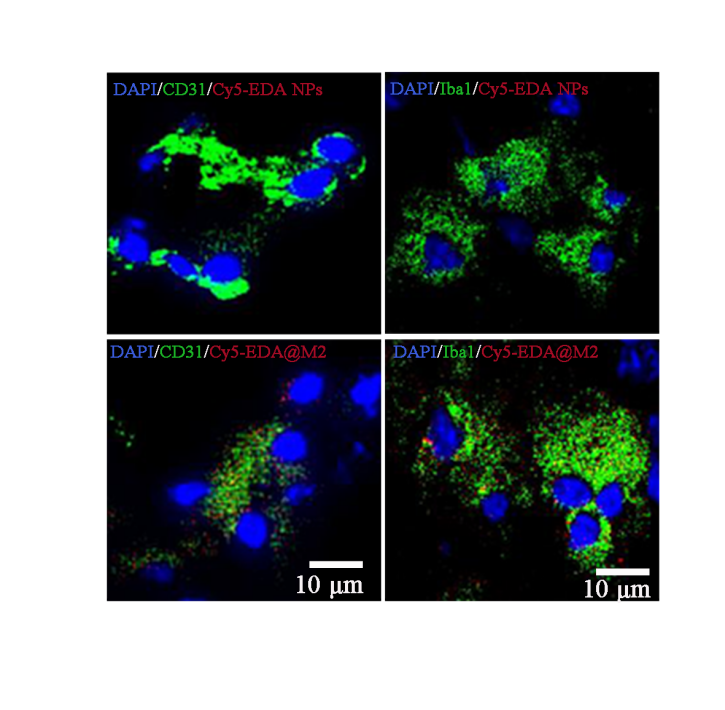


**Figure S5.** Immunofluorescence analysis of nanoparticle distribution in ischemic brain tissue. Representative brain sections stained with DAPI (nuclei, blue), CD31 (endothelial cells), Iba1 (microglia), and Cy5-labeled nanoparticles (red). Scale bar: 10 μm.

**
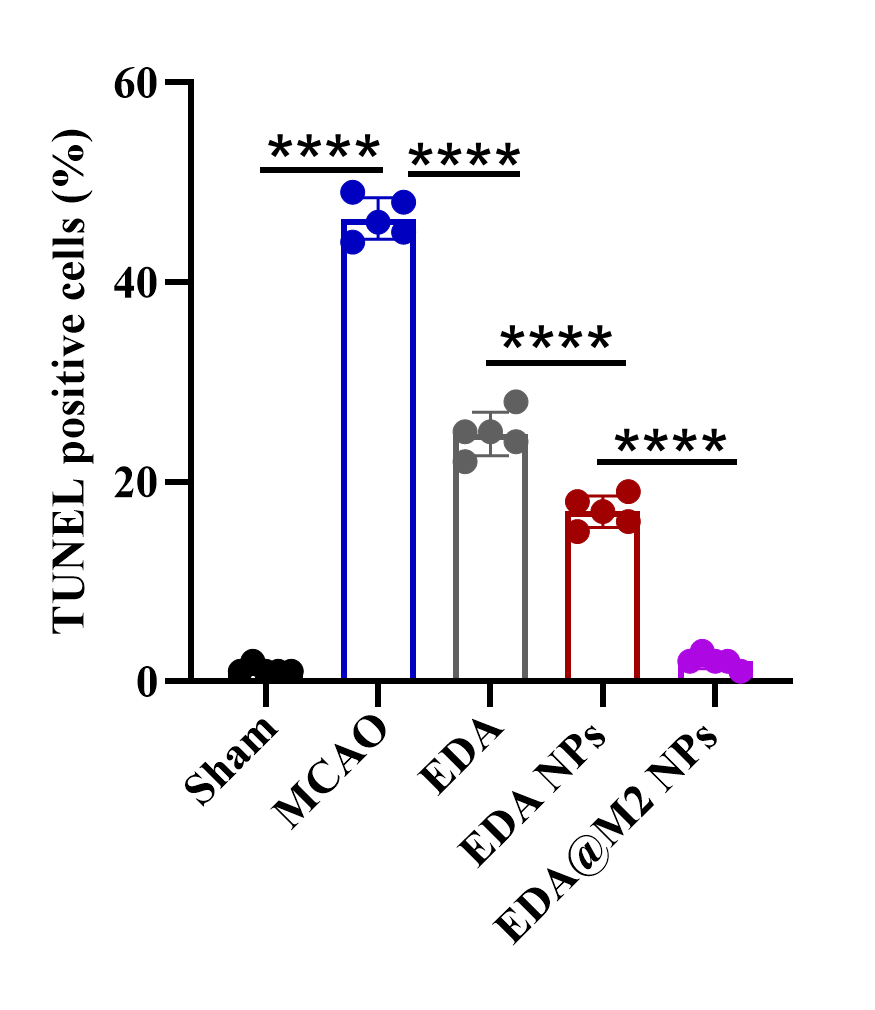
**

**Figure S6.** Quantification of in vivo neuronal apoptosis in MCAO mice following intravenous administration of PBS, EDA, EDA NPs, or EDA@M2 NPs. EDA@M2 NPs significantly reduced the percentage of apoptotic cells compared to the PBS and EDA NP groups, indicating enhanced neuroprotection. n = 5 per group. Data are presented as mean ± SD. ****p < 0.0001.


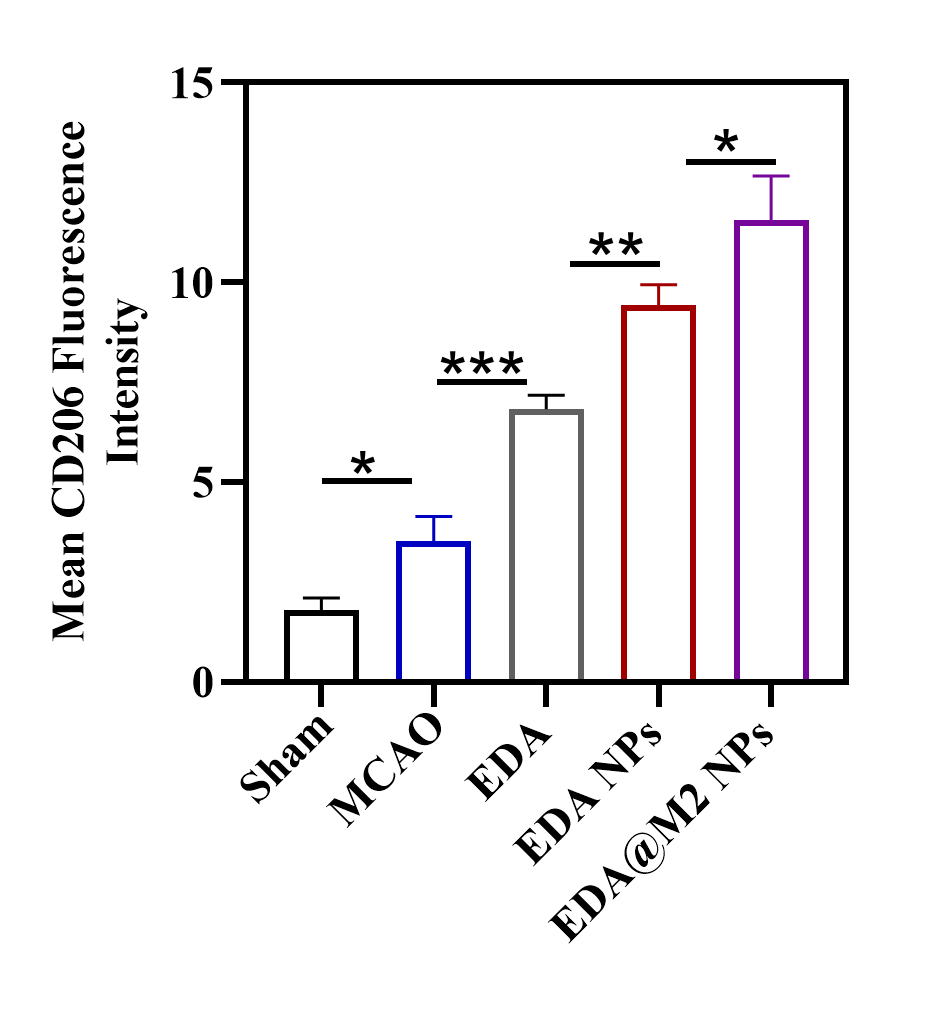

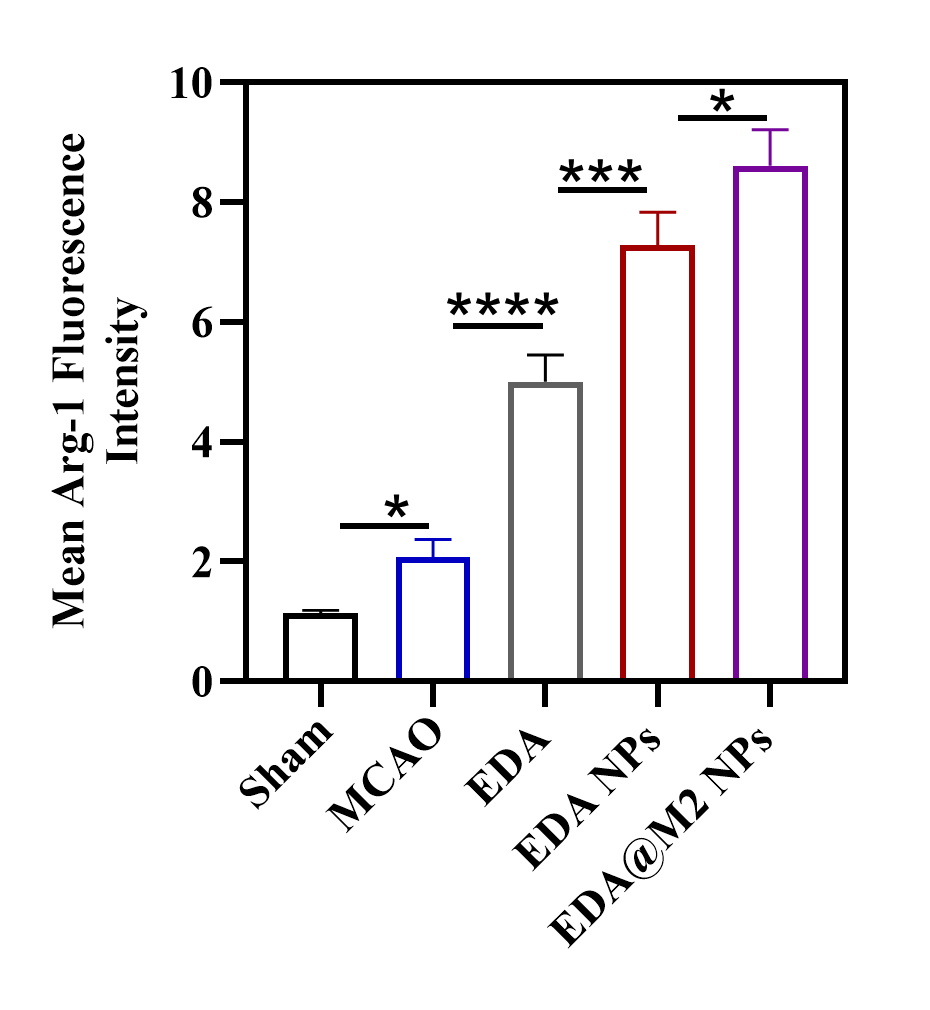

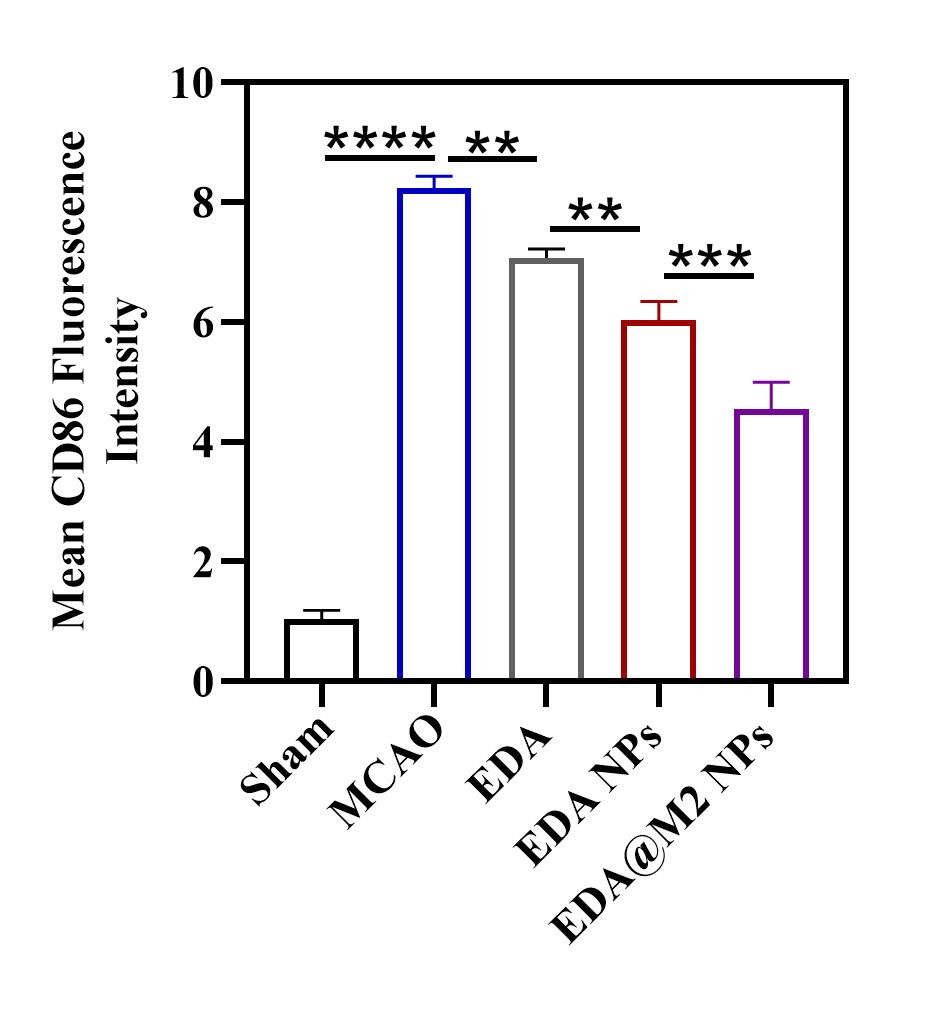


**Figure S7.** Quantitative analysis of mean fluorescence intensity (MFI) of CD86, CD206, and Arg-1 in each group. n = 5 per group. Data are presented as mean ± SD. *p < 0.05, **p < 0.01, ***p < 0.001, ****p < 0.0001.


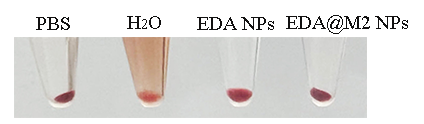

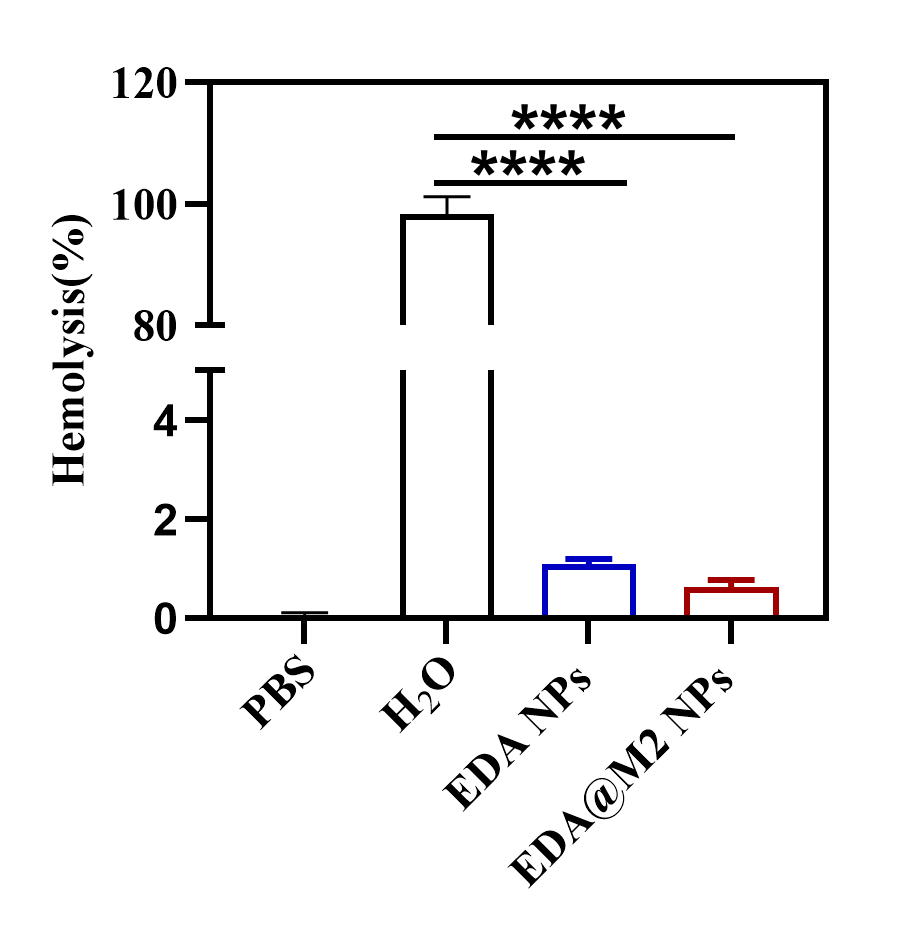


**Figure S8.** Hemolysis assay evaluating the blood compatibility of EDA@M2 NPs. Mouse red blood cells were incubated with PBS (negative control), deionized water (positive control), EDA NPs, or EDA@M2 NPs. Minimal hemolysis was observed in the nanoparticle-treated groups, indicating good hemocompatibility of EDA@M2 NPs. n = 3 per group. Data are presented as mean ± SD. ****p < 0.0001.

**Abbreviations**

Recombinant tissue-type plasminogen activator (rt-PA)

Hydrogen peroxide (H_2_O_2_)

Reactive oxygen species (ROS)

Superoxide anion (·O^2−^)

Hydroxyl radical (^.^OH)

Middle Cerebral Artery Occlusion (MCAO)

Blood-brain barrier (BBB)

Transmission electron microscopy (TEM)

Dynamic light scattering (DLS)

Confocal laser scanning microscopy (CLSM)

Lipopolysaccharide (LPS)

Enzyme-linked immunosorbent assay (ELISA)

Hematoxylin and eosin (H&E)
